# Harvesting Water‐Evaporation‐Induced Electricity Based on Liquid–Solid Triboelectric Nanogenerator

**DOI:** 10.1002/advs.202201586

**Published:** 2022-04-17

**Authors:** Jingu Chi, Chaoran Liu, Lufeng Che, Dujuan Li, Kai Fan, Qing Li, Weihuang Yang, Linxi Dong, Gaofeng Wang, Zhong Lin Wang

**Affiliations:** ^1^ Ministry of Education Engineering Research Center of Smart Microsensors and Microsystems College of Electronics and Information Hangzhou Dianzi University Hangzhou 310018 China; ^2^ China Jiliang University Hangzhou 310018 China; ^3^ School of Materials Science and Engineering Georgia Institute of Technology Atlanta GA 30332‐0245 USA; ^4^ College of Information Science & Electronic Engineering Zhejiang University Hangzhou 310027 China; ^5^ Beijing Institute of Nanoenergy and Nanosystems Chinese Academy of Sciences Beijing 101400 P. R. China

**Keywords:** electricity generation, streaming potential/current, triboelectric nanogenerators, water‐evaporation

## Abstract

Harvesting energy from natural water evaporation has been proposed as a promising alternative to supply power for self‐powered and low‐power devices and systems, owing to its spontaneous, ubiquitous, and sustainability. Herein, an approach is presented for harvesting water‐evaporation‐induced electricity based on liquid–solid triboelectric nanogenerators (LS‐TENGs), which has various advantages of easy preparation, substrate needless, and robustness. This developed harvester with porous Al_2_O_3_ ceramic sheet can generate a continuous and stable direct current of ≈0.3 µA and voltage of ≈0.7 V by optimizing the sheet physical dimensions and ambient parameters such as relative humidity, temperature, wind velocity, and ion concentration. The output power also can be improved significantly by series or parallel connection the harvesters, which has superior electrical compatibility and environmental suitability. The development of the water‐evaporation‐induced electricity harvesting shows many application prospects including power supply for digital calculator and charging capacitor. This research provides an in‐depth experimental study on water‐evaporation‐induced electricity harvesting based on LS‐TENGs and an efficient approach to supply electricity for low‐power devices.

## Graphical abstract

A liquid–solid triboelectric nanogenerator (LS‐TENG) based on a porous Al_2_O_3_ sheet is developed for harvesting water‐evaporation‐induced electricity. The optimized LS‐TENG achieves sustainable stable output direct current ≈0.3 µA and voltage ≈0.7 V. The output electricity can be multiplied to power digital calculators or charge capacitors by connecting the LS‐TENGs in series or parallel.

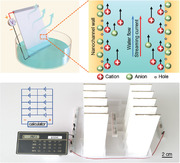

## 1 Introduction

Sustainable and clean environmental energy becomes critical to the progress of human civilization, attributing to the exhaustion of fossil energy sources and environmental deterioration.^[^ ^1^, ^2^, ^3^ ^]^ Harvesting energy from environment has significant advantages of eco‐friendliness, low‐cost and sustainability, which developed rapidly in recent decades. Many feasible energy harvesting mechanisms such as piezoelectricity,^[^ ^4^, ^5^, ^6^, ^7^ ^]^ thermoelectricity,^[^ ^8^, ^9^, ^10^ ^]^ triboelectricity,^[^ ^11^, ^12^, ^13^, ^14^, ^15^, ^16^, ^17^, ^18^, ^19^, ^20^ ^]^ and so on,^[^ ^21^, ^22^, ^23^ ^]^ have been presented to improve the output performance. Among them, harvesting natural water‐evaporation energy has gained more attention attributing to its spontaneity, ubiquity and direct current (DC) output.

Elegant approaches to optimize the performance of water‐evaporation‐induced electricity have been reported. In particular, Zhou and Wan developed a water evaporation energy harvester based on treated carbon black sheet in 2017. The hydrophilic sheet provides a capillary force and driven the water flow climbing upward. The water‐evaporation‐induced electricity generates accompanying the liquid–solid triboelectrification.^[^ ^24^ ^]^ Inspired by this work, researchers improved the performance of the generator by optimizing its materials,^[^ ^25^, ^26^, ^27^ ^]^ structure,^[^ ^28^, ^29^, ^30^, ^31^ ^]^ fabrication process^[^ ^32^, ^33^, ^34^ ^]^ or their combinations.^[^ ^2^, ^35^, ^36^ ^]^ The excellent water‐evaporation electricity nanogenerators have significantly improved its key performance indicators such as open‐circuit voltages (<2 V), short‐circuit current (<1 µA). These results manifest that the water‐evaporation‐induced electricity has a promising power supply for low consumption device. However, some deficiencies such as poor mechanical strength, lower robustness, high‐cost and complicated fabrication process should be solved to meet the requirement of complex application conditions.

Here, we develop a liquid–solid triboelectric nanogenerator (LS‐TENG) based on commercial porous Al_2_O_3_ ceramic sheet with a simple painting electrode layer. Guided by Wang model,^[^ ^37^, ^38^ ^]^ this optimized LS‐TENG achieves a continuous and stable output direct current ≈0.3 µA, voltage ≈0.7 V, which has prominent advantages of robustness, easy‐fabrication, low‐cost and stable performance. The output performance can be enhanced significantly by series or parallel connecting the LS‐TENGs attributing to its superior electrical compatibility. The robustness of ceramic enables the LS‐TENG with a better output stability and longer service life, in contrast with carbon or nanowire materials. Also, the simple fabrication process facilitates the batch production and applications. This research deeply studies on water‐evaporation‐induced electricity harvesting based on Wang model and promotes its potential application.

## 2 Methods and Experiments

### 2.1 Model and Working Principle

**Figure 1:**
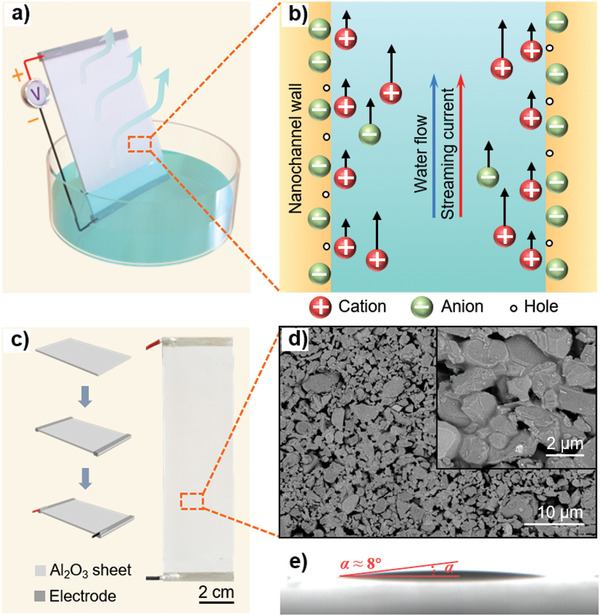
Water‐evaporation‐driven electricity generation with porous Al_2_O_3_ sheet. a) Experimental set‐up for measuring evaporation‐driven electricity. b) Schematic of the water‐flow‐induced streaming current in a single nanochannel of the sheet. c) Schematic of the LS‐TENG fabrication process and photograph of the LS‐TENG. d) SEM image of the Al_2_O_3_ sheet. e) Static contact angle of the LS‐TENG surface.

The designed LS‐TENG consists of a superhydrophilic porous Al_2_O_3_ ceramic sheet, top and bottom electrodes and copper wires. The water flow will climb along nanochannels inside the ceramic sheet to the top electrode as the bottom electrode is immersed into the water sink, which is driven by the capillary force (**Figure** **1**a,b). It reaches a pressure balance between the two ends of flow when the water pillar ascends to a certain height inside the nanochannel. Simultaneously, the fluid pillar above the water level will evaporate at the surficial nanochannels of the sheet, which generates a pressure difference between two ends of the fluid flow. The evaporated water will be supplemented by driving the water in the sink climbing upward continuously to keep the pressure balance. Consequently, a continuous ascending flow generates in the Al_2_O_3_ microfluid nanochannels. According to Wang model,^[^ ^37^, ^38^ ^]^ the molecules and ions in water flow will impact the porous Al_2_O_3_ surface when the water fluid flows inside the solid nanochannel, owing to the thermal motion and liquid pressure. Meanwhile, the electrons will transfer between the water molecules and solid atoms due to the overlap of the electron clouds of the water molecules and solid atoms. Given the AlN‐water contact electrification (CE),^[^ ^37^ ^]^ we assume that the Al_2_O_3_ gains electrons (positive charges from water) during the liquid–solid CE. Water molecule losses one electron and becomes water ion, but the water ions are not stable and quickly become two radicals following ^[^ ^39^ ^]^

(1) $$H_{2}O^{+}+H_{2}O\to \mathrm{OH}+H_{3}O^{+}$$

In addition, the ionization reaction may occur simultaneously on the solid surface, which provides anions to Al_2_O_3_. Consequently, the Al_2_O_3_ nanochannel surface will be negatively charged (Figure 1b), ascribed to the dominant role of ion transfer. Based on Wang model, the electron transfer changes the “neutral” atoms on the solid surface into ions, which is trapped in the surface state due to the impacts between liquid and solid atoms. The ionization reaction also provides “neutral” atoms with extra electrons during the liquid–solid CE. However, the extra electrons of the “neutral” atoms generated in the ionization reaction are trapped in the atomic orbitals. The potential barrier of atomic orbitals to restrain the electrons from escaping may be higher than that of the surface states. Therefore, the transferred electrons are more removable from the solid surface, in contrast with the electrons produced in ionization reactions. ^[^ ^37^ ^]^

Flowing the liquid–solid CE, the mobile cations in water will be attracted to migrate toward the negatively charged nanochannel surface due to electrostatic interactions, which forms an electric double layer (EDL).^[^ ^37^, ^38^, ^40^ ^]^ Then, the transportation of cations in EDL along with the flow will form a net positive charge transport, known as the streaming current. Meanwhile, the induced electric field created by the resulting polarization of charge distribution along the flowing axis will lead to a streaming potential.^[^ ^41^, ^42^, ^43^ ^]^

### 2.2 Fabrication of the LS‐TENG

Figure 1c shows the schematic diagram of the LS‐TENG fabrication process. First, the porous Al_2_O_3_ sheet was purchased and cleaned with the required size. The 3 mm width electrodes layers (Ag paste) were coated on the two ends of the sheet by a simple painting method or by the microelectronic flexible printer (Scientific 3A, Prtronic, China). Finally, it wired on the two electrodes and then dried in air at room temperature. The SEM and photo images of the Al_2_O_3_ sheet are shown in Figure 1c,d, which manifest the sheet composed of a mass of randomly distributed nanoparticles of roughly 300–1500 nm in diameter. These nanoparticles are packed closely together to create a large amount of nanopores, which could form nanochannels for water flow. Also, the sheet is superhydrophilic with a static contact angle ≈8° (Figure 1e).

### 2.3 Experimental Measurement System

**Figure 2:**
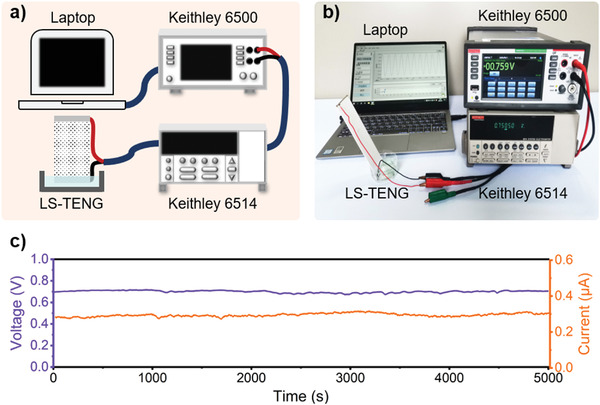
Measuring system and output performance of the LS‐TENG. a) Schematic and b) experimental measuring system of the electricity energy generated by the LS‐TENG. c) The long‐time stability of LS‐TENG output. *V*_oc_ and *I*_sc_ were generated by an LS‐TENG with the size of 40×130×1 mm^3^ at room relative humidity ≈50.0% and temperature ≈27.0 °C during the test.

**Figure** **2**a,b shows a bench system for measuring the output open‐circuit voltage *V*_oc_ and short‐circuit current *I*_sc_ of the LS‐TENG (Video S1). The bottom electrode is completely immersed into DI water sink during the test. A Keithley 6514 electrometer and a Keithley 6500 multimeter are employed to measure the output performance. Thus, the real‐time output voltage and current data can be uploaded to laptop by a special data processing software. A GDWJS‐500 programmable constant temperature and humidity test chamber is employed for the experiments on ambient temperature or relative humidity. A Testo 605i humidity and temperature meter and a Testo 405‐V1 anemometer are using for measuring relative humidity, temperature and air flow velocity, respectively. A Sartorius PB‐10 pH meter is using for measuring solution pH value. The sheet hydrophilcity is measured by a PZ‐200SD contact angle meter.

## 3 Results and Discussion

The as‐fabricated LS‐TENG with the size of 40×130×1 mm^3^ is tested by the designed measuring system. Based on the working principle, the bottom electrode is immersed in DI water. The water flow promptly climbs along the sheet attributing to its super‐hydrophilicity. And the evaporation above the water level will drag the fluid flow climbing continuously. The liquid–solid CE and climbing induces a streaming current in the ceramic sheet, which generates a potential difference between the two electrodes of LS‐TENG. Figure 2c shows the long‐term stability of output electricity even after 5000 seconds, which has a higher direct open‐circuit voltage ≈0.7 V and short‐circuit current ≈0.3 µA tested by the electrometer at room temperature ≈27.0 °C and relative humidity ≈50.0%. The slight fluctuations of the measured voltage and current were mainly caused by air flow, temperature, humidity, etc.

**Figure 3:**
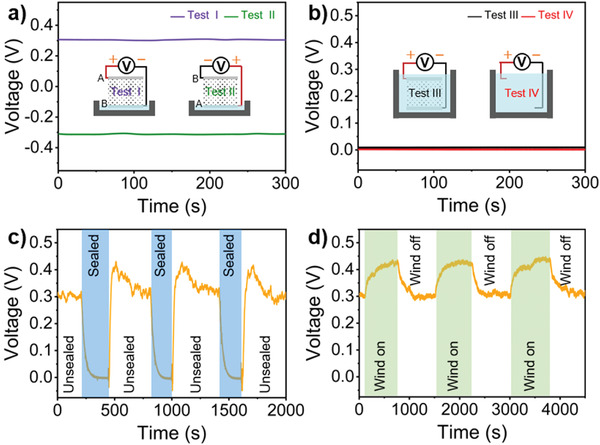
Induced electricity originating from natural water evaporation. a) *V*_oc_ generated by the device before (test I) and after (test II) the device was turned upside down. b) Test III: *V*_oc_ of the device completely submerged by water. Test IV: *V*_oc_ between two wires with the other ends immersed in water. c) *V*_oc_ of the device when the sink was periodically sealed and unsealed. d) *V*_oc_ of the device when the ambient air flow of 0.1 m s^–1^ was periodically turned on and off.

To explicitly confirm the electricity generation mechanism, we carry out four contrast experiments, such as test I–IV (**Figure** **3**a,b). For the switching‐polarity measurement in test I–II, the output voltages of LS‐TENG have a reverse polarity and equivalent value, which indicates the polarity of the voltage determined by the electrode position rather than the electrode materials. And for last two contrast experiments, there is no output voltage between the electrometer probes completely submerged by water whether or not connecting the LS‐TENG electrodes. The submerging stops the evaporation and water flow. Consequently, there is no output voltage in test III. From the four tests, we can conclude that the principle of output electricity is not primary battery effect but the liquid–solid triboelectric nanogenerator induced by the water evaporation.

To illustrate the significance of water evaporation in the sheet, we designed other contrast experiments, such as the water sink sealed or not, wind on/off. Figure 3c and Figure S1 (Supporting Information) show that the output voltages vary distinctly with the LS‐TENG sealed or not. The humidity inside the sink will rise promptly from the ambient humidity (≈55.0%) to the saturated humidity (≈100%) if it is sealed, which suppresses the water evaporation severely. Simultaneously, the output voltage decline rapidly near to 0 V. Inversely, the output voltage will restore to 0.3 V immediately if it unseals the LS‐TENG, which reveals the crucial contribution of a lower ambient humidity to the evaporation and output electricity. Sensibly, a higher air flow can also promote the water evaporation. The wind on/off experiment shows that a 0.1 m s^–1^ air flow can raise the voltage from 0.3 V to 0.4 V, which attributes to the enhanced evaporation (Figure 3d). On the contrary, the voltage will drop and return to the original 0.3 V accompanying the wind off. The periodic variation in Figure 3c,d indicate that the influence of relative humidity or wind on the output voltage of the LS‐TENG sheet is reversible. The strong correlation between the induced voltage and water evaporation demonstrates that the electricity can be harvested from water evaporation based on liquid–solid triboelectric nanogenerator.

To further optimize the output performance, we analyze the influence of the physical dimension of the LS‐TENG sheet. Based on the working principle, the output voltage generate from the evaporation‐induced water flow and CE. The width, thickness and height of the sheet have an influence on the water flow velocity, volume, etc, which impacts the output performance.

**Figure 4:**
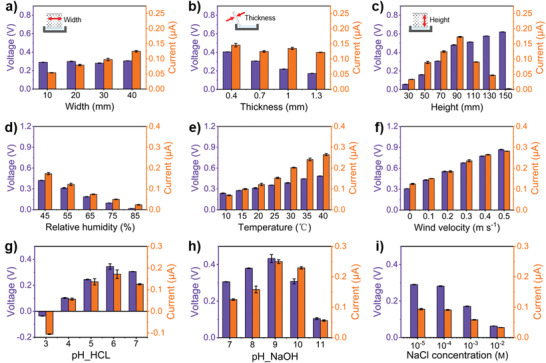
The effect of geometric dimensions, ambient conditions and ionic concentration on the LS‐TENG output performance. Variation of *V*_oc_ and *I*_sc_ with a) different sheet width (height: 70 mm, thickness: 0.7 mm), b) different thickness (height: 70 mm, width: 40 mm), c) different height between two electrodes (width: 40 mm, thickness: 0.7 mm), d) different relative humidity (20.0 °C, no wind), e) different temperature (55.0%, no wind), f) different wind velocity (55.0%, 20.0 °C), g) HCl, h) NaOH solutions of different pH value, and i) NaCl solution of different concentration. The tests in panels (g)–(i) were conducted under room condition of relative humidity ≈55.0%, temperature ≈20.0 °C, and wind velocity ≈0 m s^–1^. These tests in panels (d)–(i) were all carried out with the same sized LS‐TENG of 70 × 40 × 0.7 mm^3^.

Here, four different LS‐TENG sheet widths (10, 20, 30, and 40 mm) are selected to optimizing the physical dimension. And the output voltage and current are measured under the same ambient humidity (55.0%), temperature (20.0 °C), and wind speed (0 m s^–1^) to ensure the consistency of the experiments. The water evaporates mainly on the surficial vicinity nanochannels of LS‐TENG sheet. The total volume flow rate *Q*_st_ has a positive correlation with the sheet surficial area and water evaporation rate. The broadened width will increase the evaporating area and total volume flow rate *Q*_st_, which enhances the output current as expressed in Equation S2 (Supporting Information) and **Figure** **4**a. While the total streaming potential *V*_st_ remains almost invariable owing to the vertical parallel nanochannels (Equation S2, Supporting Information).

And for the increased thickness, it multiplies the internal nanochannels of the LS‐TENG sheet with little effect on the surficial evaporation area and rate. Consequently, *Q*_st_ and *I*_sc_ are almost invariable. There is scarcely any water streaming in the increased internal nanochannels due to the surficial evaporation. Inversely, it increases the parallel connection nanochannels and resistances. Therefore, the output voltage declines slightly as measured results in Figure 4b.

Besides the sheet width and thickness, we also explore the impact of sheet height on the output property of LS‐TENG. A LS‐TENG with a higher sheet under the range of 30–90 mm, enlarges directly the evaporating area and nanochannel length *l*, expediting the total volume flow rate *Q*_st_. Thus, the output current and voltage are improved significantly. However, the limited capillary force cannot drive the water flows in all nanochannels to climb higher than 90 mm. The insufficient water flows (at height of 90–150 mm) aggravates the internal resistance of the LS‐TENG, and decline the output current. While, the increment of *Q*_st_ will promote the potential difference between the top and bottom electrodes (Figure 4c).

The generating electricity of the LS‐TENG depends mainly on the water evaporation. Besides physical dimension, the output performance of the LS‐TENG will be definitely affected by the environmental factors such as relative humidity, temperature, wind velocity and ionic concentration. To improve the output electricity, we first carry out the relative humidity experiment in a standard test chamber with adjustable temperature and humidity. The selected relative humidities are of 45%, 55%, 65%, 75%, and 85% respectively. The measured results show that both *V*_oc_ and *I*_sc_ of the LS‐TENG decline gradually with the increasing of relative humidity (Figure 4d and Figure S2b, Supporting Information), which ascribes to the evaporation suppressed by a higher relative humidity. And for the ambient temperature and wind velocity, the output performances improves obviously, which are proportional to the temperature increasing from 10 to 40 °C (Figure 4e) and to the wind velocity boosting from 0 to 0.5 m s^–1^ (Figure 4f). The enhanced ambient temperature and wind velocity promote the water evaporation directly, and further improving the output electricity.

To explore the effect of ionic concentration on the LS‐TENG, we employ different ions solutions. For the acid and alkaline solutions tests, *V*_oc_ and *I*_sc_ increase first and then decreases with increasing of the ionic concentration (Figure 4g,h). But for the NaCl solution, *V*_oc_ and *I*_sc_ decline monotonically with increasing the concentration from 10^−5^ to 10^−2 ^mol L^–1^ (Figure 4i). For the HCl, NaOH solutions, the improved output is attributed to enhancing the ion transfer process during the liquid–solid CE under the lower ion concentration. While, the further increase of the ion concentration interferes with the electron transfer process due to the screen effect of the excessive free ions. In addition, a lower pH in acid solutions can reverse the *V*_oc_ polarity, because the modification of surface functional groups can alter the polarity of the triboelectric charges between the Al_2_O_3_ surface and aqueous solution. With the increased pH value, the Al_2_O_3_ surface is more likely to be charged negatively by absorbing hydroxide ions and obtaining electrons from water molecules.^[^ ^37^, ^38^ ^]^ But for the NaCl, the solution flow including water and NaCl molecules will climb upward. In our supposing, the concentration of NaCl solution in the Al_2_O_3_ sheet above the water level will increases continuously accompanying the water molecules evaporation, which approaches to the saturation same as the concentration of NaCl solution in the sink. And then, NaCl molecule is no longer climbing upward in the flow to balance the concentration of NaCl solution both in the Al_2_O_3_ sheet and in the sink. Finally, the concentration both in the Al_2_O_3_ sheet and in the sink will ultimately approaches to be identical for each solution (HCl, NaOH, and NaCl). The output performance will be decided by the property and concentration of solution itself. Increasing the concentration of NaCl solution leads to the excessive free ions in the flow, which can interfere with the electron transfer process due to the screen effect.^[^ ^38^ ^]^ Thus, the output performance of LS‐TENG has a remarkable declining as shown in Figure 4i.

**Figure 5:**
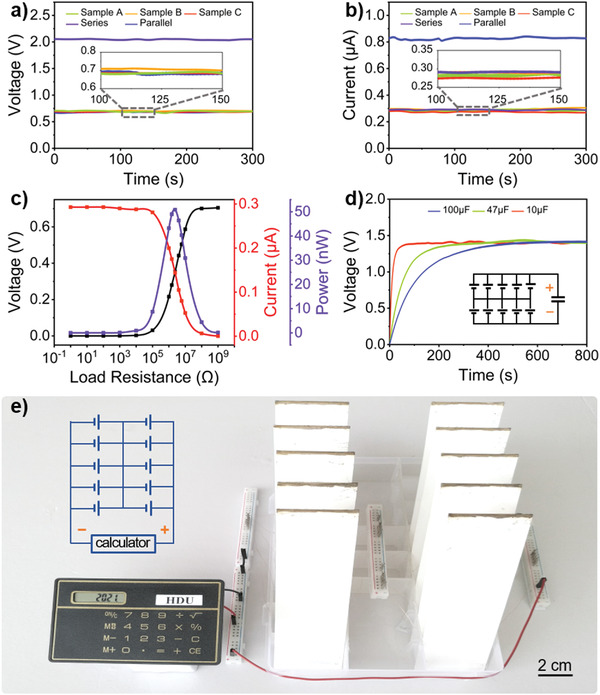
Output performance and applications of the LS‐TENG. a) *V*_oc_ and b) *I*_sc_ of an individual sample, and three samples in series or parallel. c) Output voltage, current, and power of the LS‐TENG with different load resistances. d) The LS‐TENGs charging the capacitors of 100, 47, and 10 µF. Inset: circuit diagram. e) Photograph of the LS‐TENGs powering an electronic calculator. Inset: circuit diagram. All experiments in this figure were conducted with LS‐TENGs sized 40×130×1 mm^3^ at room relative humidity ≈50.0% and temperature ≈27.0 °C.

As a power supply, improving the output performance is crucial to its applications. Here, we scale up the output electricity by simply connecting multiple LS‐TENGs in series or parallel. **Figure** **5**a,b shows the experimental results that *V*_oc_ has been boosted nearly triple by connecting the three LS‐TENGs in series, as well as *I*_sc_ in paralleling connection. Also, the output performance of our device is investigated by connecting external loads with different resistances. Figure 5c show that the voltage increases from nearly zero to 0.7 V with the load resistance increasing from 1 Ω to 1 GΩ. Accordingly, the current decreases from ≈0.3 µA to nearly zero, which achieves a maximum output power of 50.8 nW with load of ≈2.4 MΩ. The optimized LS‐TENGs have many potential applications attributing to its stable and high direct current output. Figure 5d,e shows that the integrated ten LS‐TENGs connected in series and parallel can charge commercial capacitors for energy storage, and power an electronic calculator sustainably. For different capacitors (100, 47, and 10 µF), the LS‐TENGs can charge them to 1.4 V (Videos S2, Supporting Information). Also, it has a power ability for an electronic calculator with multiple operations continuously (Videos S3, Supporting Information). All the experimental results indicate that the fabricated LS‐TENG based on porous Al_2_O_3_ sheet shows superior electrical compatibility and stability.

## 4 Conclusion

In summary, we present an approach for harvesting water‐evaporation‐induced electricity based on liquid–solid triboelectric nanogenerator (LS‐TENG). Guided by Wang model, this optimized LS‐TENG consists of commercial porous Al_2_O_3_ ceramic sheet, and achieves sustainable stable DC electricity output with *V*_oc_ of ≈0.7 V and *I*_sc_ of ≈0.3 µA. The output power can be multiplied by simply connecting LS‐TENGs in series or parallel, which has superior electrical output performance, compatibility, robustness and environmental suitability. These excellent capabilities enable the LS‐TENG to many practical applications such as power supply for digital calculator and charging capacitor. Our endeavor presents not only an in‐depth experimental exploration on water‐evaporation‐induced electricity harvesting, but also a new perspective to the application of LS‐TENG mechanism.

## Conflict of Interest

The authors declare no conflict of interest.

## Supporting information

Supporting Information

Supplemental Video 1

Supplemental Video 2

Supplemental Video 3

## Data Availability

The data that support the findings of this study are available from the corresponding author upon reasonable request.
