# Supplementary material for: Harvesting Water‐Evaporation‐Induced Electricity Based on Liquid–Solid Triboelectric Nanogenerator
Source: Adv Sci (Weinh). 2022 Apr 17;9(17):2201586. doi: 10.1002/advs.202201586 (PMC9189656; doi:10.1002/advs.202201586)
Supplement: Supplementary file 1 — Supporting Information [file ADVS-9-2201586-s001.pdf]

ADVANCED SCIENCE
Open Access

Supporting Information

for *Adv. Sci.*, DOI 10.1002/advs.202201586

Harvesting Water-Evaporation-Induced Electricity Based on Liquid–Solid Triboelectric Nanogenerator

Jingu Chi, Chaoran Liu, Lufeng Che, Dujuan Li, Kai Fan, Qing Li, Weihuang Yang, Linxi Dong*, Gaofeng Wang* and Zhong Lin Wang**

Supporting Information

Harvesting Water-Evaporation Induced Electricity Based on Liquid-Solid Triboelectric Nanogenerator

*Jingu Chi, Chaoran Liu**, *Lufeng Che, Dujuan Li, Kai Fan, Qing Li, Weihuang Yang, Linxi Dong**, *Gaofeng Wang* *, *Zhong Lin Wang* *

1. The streaming current and potential mechanism

To further illuminate the energy harvesting mechanism, we assume the porous Al_2O_3 sheet consisting of multiple vertical parallel nanochannels (capillary tubes). First, the water flow climbs upwards to a certain height inside the nanochannel driven by the capillary force. Then, the height of the water flow declines, owing to the evaporation of water on the sheet surface. Consequently, a pressure difference ΔP occurs between the two ends of fluid flow inside the nanochannel. The pressure difference will drive the flow climb upwards again. Therefore, sustained evaporation will lead to continuous upward water flow. In another word, the water flow rate depends on the evaporation rate. The water evaporates mainly on the surface of LS-TENG sheet, which supplied by the water flow in the surficial vicinity nanochannels. Thus, we focus on the surficial nanochannels flow. The surficial nanochannels water flow forms an EDL at the liquid-solid interface. For a single nanochannel filled with water, the net positive charges transport in upward water flow forms a streaming current I_{ss}. Meanwhile, the induced electric field created by the resulting polarization of charge distribution along the flowing axis leads to a streaming potential V_{ss}.[1,2] The I_{ss} and V_{ss} can be given as[3,4]

$$I_{ss} = \frac{\varepsilon_0 \varepsilon_r \zeta A}{\eta l} \Delta P$$
$$V_{ss} = \frac{\varepsilon_0 \varepsilon_r \zeta}{\sigma \eta} \Delta P \qquad \text{(S1)}$$

where ζ is the zeta potential, ΔP is the water pressure difference between two ends of a nanochannel. ε_r, η, σ, l and A are water relative permittivity, viscosity, conductivity, nanochannel length and cross sectional area, respectively. According to Hagen−Poiseuille equation, the volume flow rate inside the single nanochannel can be expressed as $Q_{ss} = A^2 \Delta P / 8\pi\eta l$.[4] The total streaming current I_{st}, potential V_{st} and flow rate Q_{st} of the LS-TENG can be considered as parallel connection of all the surficial vicinity nanochannels.

Therefore, $I_{st} = nI_{ss}$, $V_{st} = V_{ss}$ and $Q_{st} = nQ_{ss}$, where n is the amount of surficial nanochannels. Then, we can express I_{st} and V_{st} as follow:

$$I_{st} = \frac{8\pi\varepsilon_0\varepsilon_r\zeta}{A} Q_{st}$$
$$V_{st} = \frac{8\pi\varepsilon_0\varepsilon_r\zeta l}{\sigma A^2} \frac{Q_{st}}{n} \qquad (S2)$$

2. Measuring the LS-TENG sealed in a sink

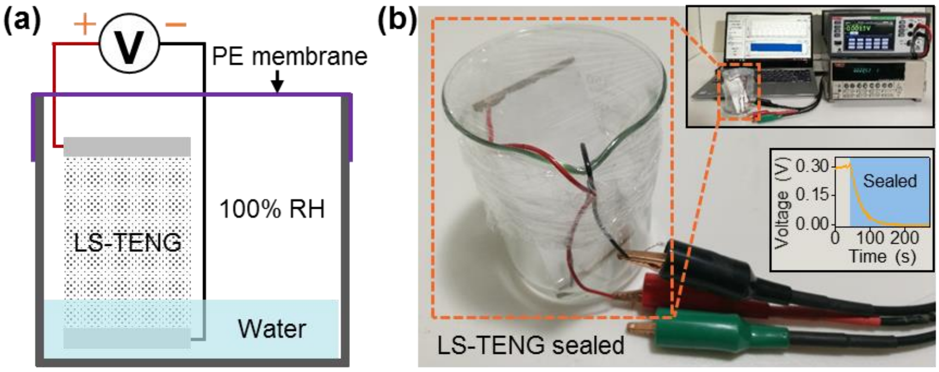

Figure S1. Measuring the LS-TENG sealed in a sink. (a) Schematic and (b) experimental photograph of measuring the LS-TENG sealed.

3. The real-time output voltage and current

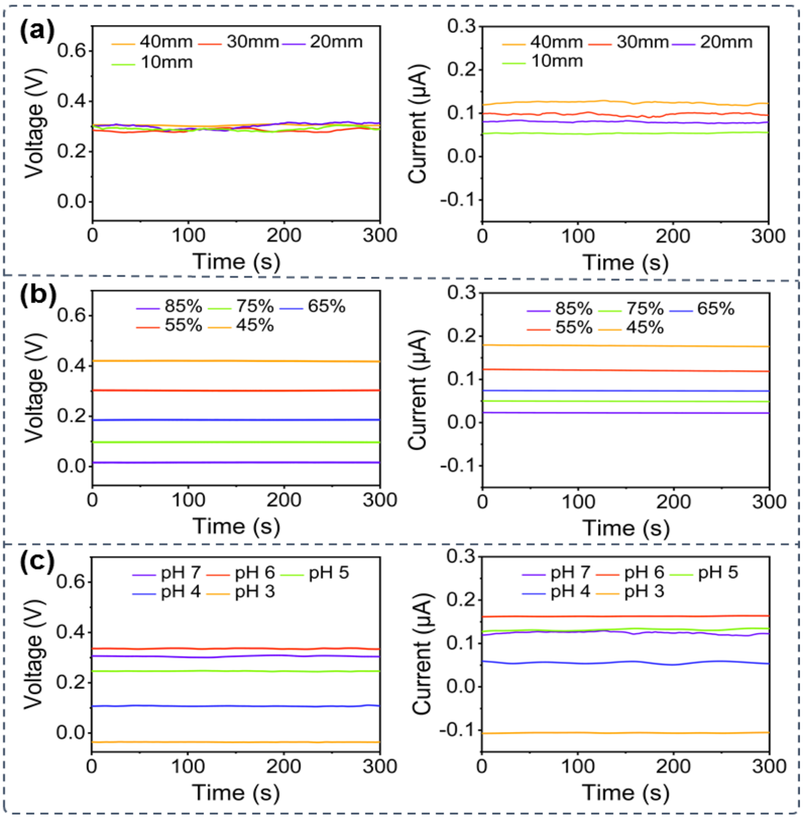

Figure S2. The real-time voltage V_{oc} and current I_{sc} under different conditions such as (a) width, (b) relative humidity and (c) pH.

4. The robustness and mechanical strength of the LS-TENG

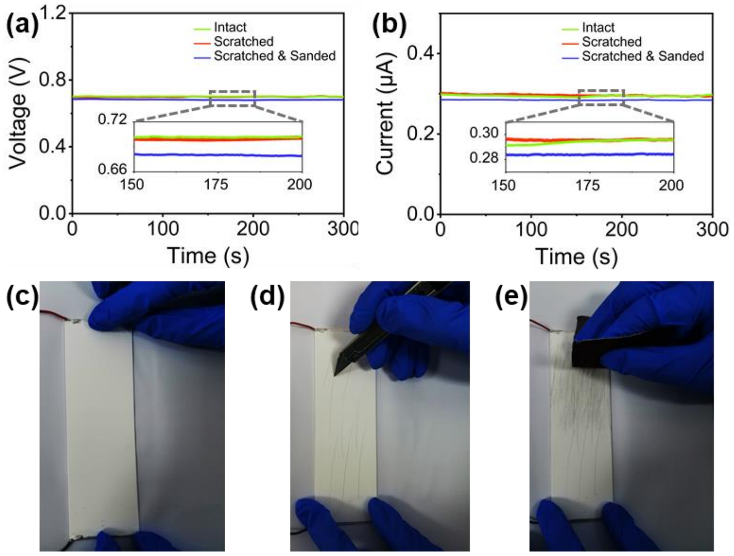

Figure S3. The robustness and mechanical strength test experiments. (a) Output voltage. (b) Output current. (c) Intact LS-TENG. (d) Scratched LS- TENG. (e) Scratched and Sanded LS- TENG.

5. Rising process of output voltage and current of LS-TENG

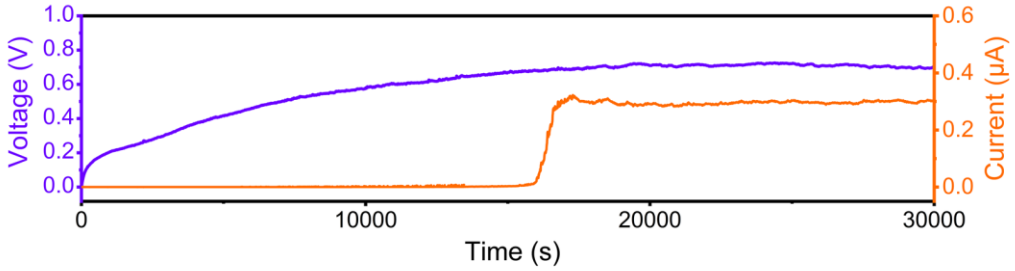

Figure S4. The rising process of output voltage and current of LS-TENG.

6. Absorption water rate of LS-TENG

We have supplemented the experiment to characterize the water delivery efficiency of commercial porous Al_2O_3 ceramic sheet by the weighing method. The absorption water process of the Al_2O_3 sheet is shown in Figure S5. Firstly, we weight the dry Al_2O_3 sheet with the size of 130×40×1 mm3. The bottom electrode is immersed into water in an unsealed sink. Then, the immersed Al_2O_3 sheet will be weighted every ~ 30 minutes. The experiment was carried out at the room temperature ~ 11.4 °C and relative humidity ~ 55.8 %. Also, we can obtain the average absorption rate as 6.57 mg/min.

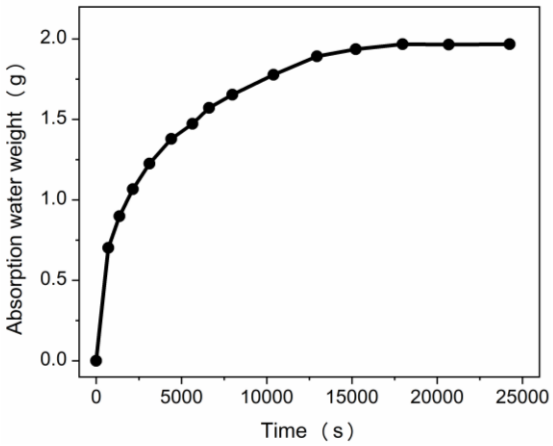

Figure S5. Absorption water weight in the Al_2O_3 sheet.

Video legends

Video S1. Measuring the output voltage of a LS-TENG

Video S2. Charging a capacitor with ten LS-TENGs connected in series and parallel

Video S3. Powering a calculator with ten LS-TENGs connected in series and parallel

References

[1] R. Zhang, S. Wang, M.H. Yeh, C. Pan, L. Lin, R. Yu, Y. Zhang, L. Zheng, Z. Jiao, Z.L. Wang, Adv. Mater. **2015**, 27, 6482.

[2] J. Yang, F.Z. Lu, L.W. Kostiuk, D.Y. Kwok, J. Micromech. Microeng. **2003**, 13, 963.

[3] W. Olthuis, B. Schippers, J. Eijkel, A. van den Berg, Sens. Actuators, B **2005**, 111, 385.

[4] X.B. Zhou, W.L. Zhang, C.L. Zhang, Y. Tan, J.C. Guo, Z.N. Sun, X. Deng, ACS Appl. Mater. Interfaces **2020**, 12, 11232.
